# Adaptive respiration patterns by antlion larvae support the chthonic hypothesis for discontinuous gas exchange cycles

**DOI:** 10.1016/j.isci.2026.117638

**Published:** 2026-09-24

**Authors:** Matthew S. Lehnert, Kendall O. Myers

**Affiliations:** 1 Department of Biological Sciences, Kent State University at Stark, North Canton, OH 44720, USA

**Keywords:** insect respiration, hypoxic-hypercapnic environment, DGC, hygric hypothesis, insect trachea

## Abstract

Insects display various respiration patterns using a respiratory system composed of valve-like spiracles and branching conduit-like tracheae. However, the adaptive value of discontinuous gas exchange cycles (DGCs), a respiratory pattern with unique gas exchange sequences, remains unanswered. Here, we used flow-through respirometry to study respiration patterns of antlion larvae, *Myrmeleon crudelis*, an insect that resides in pits constructed in soil, a possibly hypoxic-hypercapnic environment. Exposed antlions displayed continuous respiration patterns; however, antlion larvae inside pits switch to DGC, despite no changes in environmental parameters. Furthermore, transpiration rates did not differ between treatments. Investigations with confocal and scanning electron microscopy of tracheae revealed a hydrophobic chemistry that likely reduces water vapor permeability from hemolymph and an inner hierarchical topography that would enable efficient gas exchange. These findings show that the occurrence of DGCs in *M. crudelis* is associated with burrowing underground, suggesting it is a respiratory adaptation for hypoxic-hypercapnic subterranean environments.

## Highlights

• Antlion larvae use DGC respiration when in self-constructed subterranean pits
• Outside of the pits, exposed larvae use continuous respiration patterns
• The overall gas exchange and transpiration rates were similar in both treatments
• Respiration patterns might be adaptive to hypoxic-hypercapnic environments

## Graphical abstract

**Figure:**
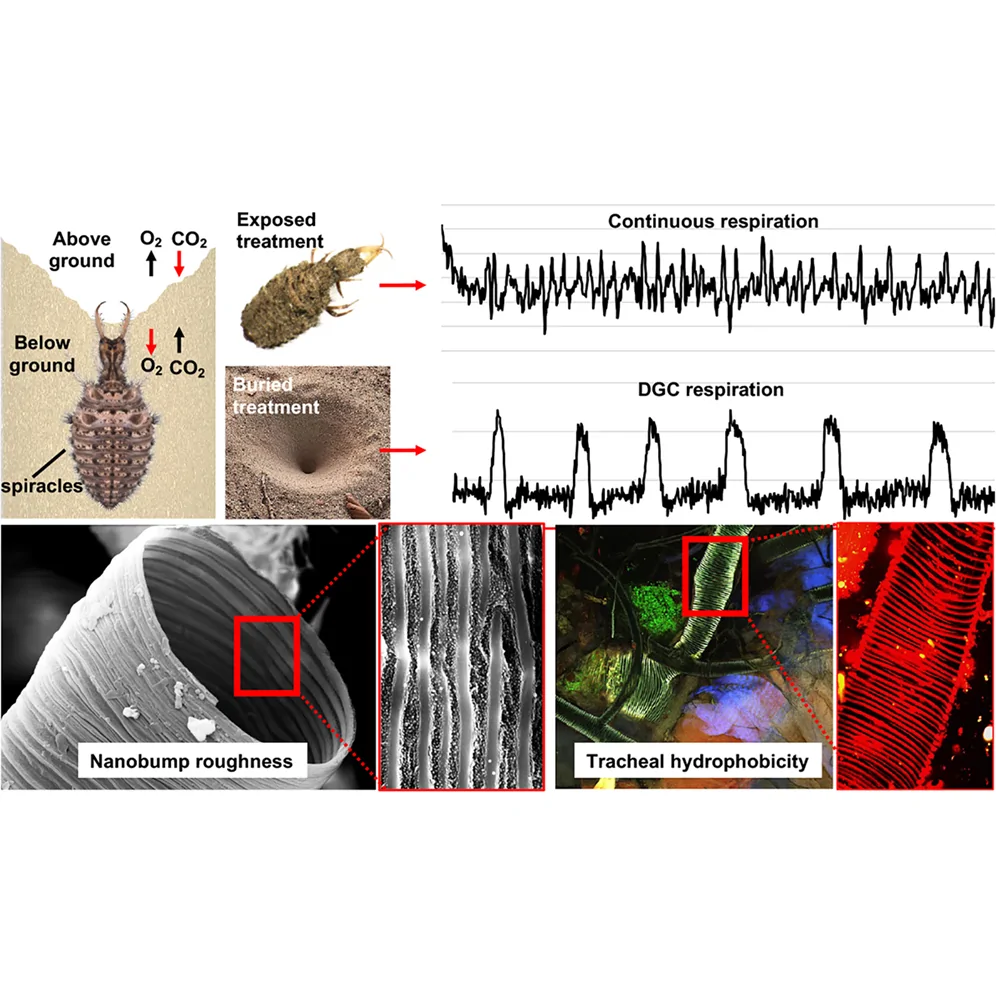

## Teaser

Environmental science; Environmental chemistry; Environmental fluid dynamics; Relation between agriculture and environment

## Introduction

The respiratory system of insects serves as a key innovation contributing to their enormous diversification and global occupancy. Respiratory diffusive airflow occurs in an intricate network of cuticle-lined and conduit-like tracheae, which extend from valve-like spiracles on the lateral sides of the abdomen and thorax. The tracheae continue to branch into smaller conduits until reaching micro-sized terminal ends, the liquid-filled tracheoles, which deliver O_2_ directly to tissues and allow CO_2_ to escape by the same pathway.^1^^,^^2^^,^^3^^,^^4^ Insects can exhibit different respiratory patterns, including cyclic, continuous, and discontinuous gas exchange cycles (DGCs) that relate to life history traits, phylogeny, environmental factors, and a deep evolutionary history that spans 400 million years.^5^^,^^6^^,^^7^

DGC, the most puzzling of the respiratory patterns, are characterized by sequential and cyclical phases of gas exchange caused by actions of the spiracles. During the flutter phase (*F*), spiracles rapidly open and close allowing O_2_ to enter the trachea but lead to a buildup of CO_2_ in the tracheal system. Once CO_2_ levels reach a critical threshold,^8^ the spiracles fully open (open phase, *O*) and a burst of CO_2_ exits the trachea. The spiracles then close (closed phase, *C*), which limits gas flow.^9^^,^^10^

Several hypotheses have been proposed about the evolutionary origins and significance of DGC,^11^ but current evidence for the historically popular hygric hypothesis (water conservation), is contentious.^12^^,^^13^ Alternatively, the chthonic hypothesis emphasizes DGC respiratory patterns evolved in response to hypoxic-hypercapnic (low O_2_-high CO_2_) environments,^11^ such as subterranean habitats. Testing the chthonic hypothesis, however, is particularly challenging due to the need of measuring respiratory patterns of subterranean insects while under experimental conditions that parallel their natural life history traits.

Here, we addressed this challenge by studying the respiratory patterns of larvae of the antlion, *Myrmeleon crudelis*, a neuropteran species that resides at the bottom of self-constructed pits. Antlion larvae dig conical-shaped pits in sandy soil by moving backwards in concentric circles, using their shovel-like heads as a tool to throw out sand particles as they make their way deeper into the pit.^14^^,^^15^^,^^16^ The larvae sit at the bottom of the pits, where they remain for up to 3 years, with their body and spiracles completely covered in substrate, and only the mouthparts exposed, ready to capture prey that falls into the pit.^17^^,^^18^^,^^19^ Although the spiracles of antlions are only a few millimeters below the soil surface, O_2_ concentration levels can rapidly decrease in soil that comprises small particle sizes, such as the sandy soils preferred by antlions for pit building,^20^ where O_2_ diffusive rates decrease due to the low soil porosity.^21^ Furthermore, moistened soil strongly affects O_2_ diffusion rates, contributing to soil hypoxia.^22^ The unique life history traits of antlion larvae provide an excellent model for testing the validity of the chthonic hypothesis, and this was exploited here by measuring respiration rates of exposed antlions and those inside pits via flow-through respirometry. Our findings are further interpreted with insights of tracheal morphology and wettability, and general patterns in transpiration rates.

## Results

### Respiration patterns of antlion larvae differ between exposed and buried treatments

Larvae of *M. crudelis* placed in the exposed treatment (no pit, *n* = 5) displayed a continuous respiration pattern, where there were persistent fluctuations in CO_2_ output (Figure 1A). During the recordings, individuals of *M. crudelis*, though not secured inside the respirometer, remained stationary for the 30-min period. The average CO_2_ output of larvae in the exposed treatment was 9.81 ± 0.03 μg g^−1^ min^−1^.

**Figure 1:**
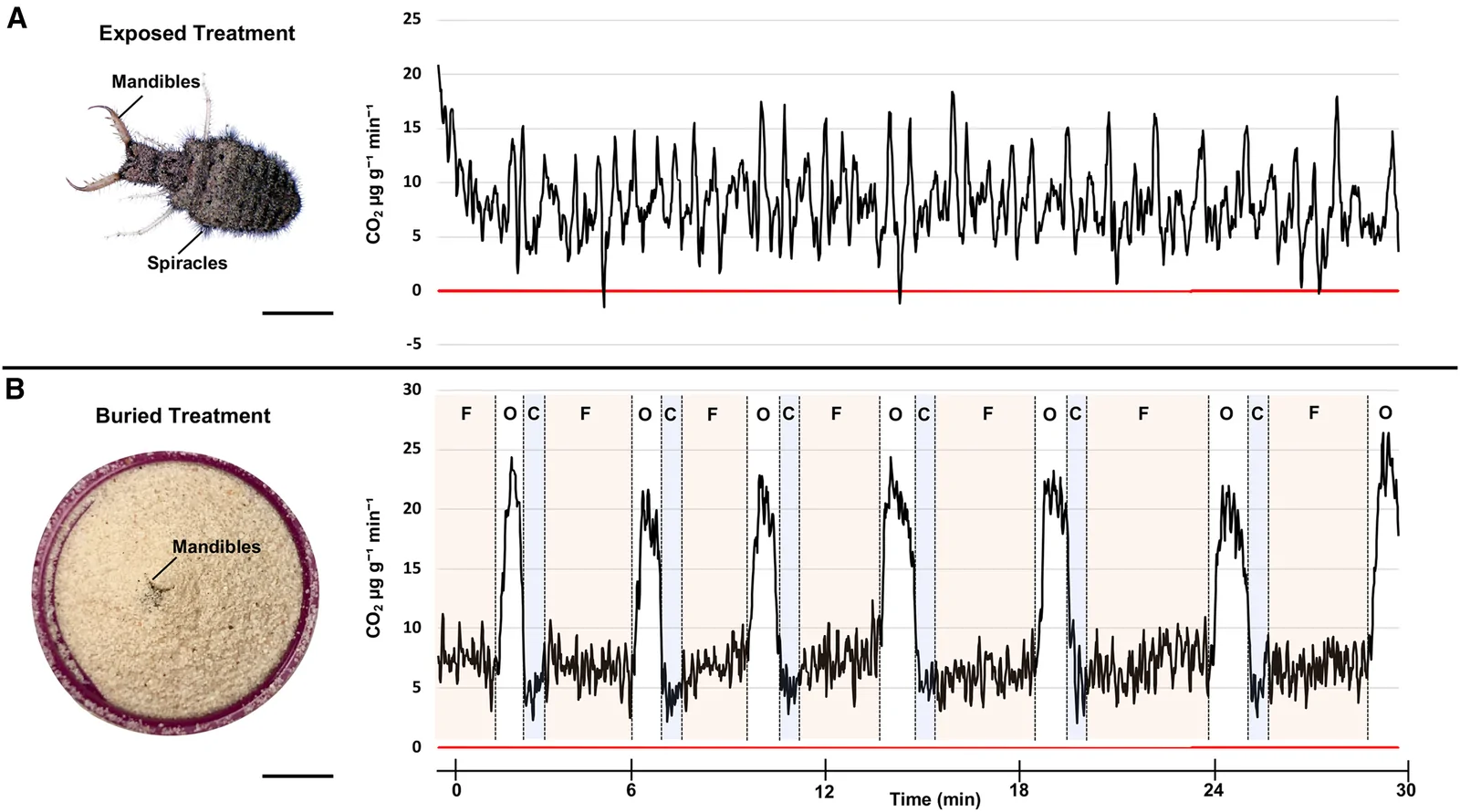
Gas exchange patterns among antlion larvae in the exposed and buried treatments (A) Larvae in the exposed treatment (no pit) expressed continuous respiratory patterns. (B) Larvae in the buried treatment (inside the self-constructed pit) revealed DGC respiratory patterns that included the flutter (*F*), open (*O*), and closed (*C*) phases. Scale bars: (A) 1 mm and (B) 4 mm.

When placed in the buried treatment (inside pit, *n* = 5), *M. crudelis* displayed a DGC respiratory pattern where three distinct repeating phases were observed: the *F*, *O*, and *C* phases (Figure 1B). We found that the *F* phase (145.88 ± 9.02 s) was significantly longer than the other phases (*C* phase, 63.81 ± 6.15 s; *O* phase, 79.84 ± 2.96 s) (ANOVA, F_(2,14)_ = 59.17, *p* < 0.0001). CO_2_ measurements were recorded during the *C* phase; however, these values were low with smaller fluctuations than the other phases. Antlions in the buried treatment had an average CO_2_ output of 9.18 ± 1.5 μg g^−1^ min^−1^. Interestingly, the overall average CO_2_ output did not significantly differ between treatments (*t* test, t_(8)_ = 0.39, *p* = 0.36); therefore, it is only the respiration patterns that differ, not the overall amount of expelled CO_2_.

### Transpiration rates of antlion larvae are similar between tested treatments

Although there were no changes in precipitation or relative humidity to induce the changes in respiration patterns, we examined transpiration rates from larvae in each treatment to determine if DGC patterns reduce water loss, as predicted by the hygric hypothesis. We found no significant differences in the average transpiration rates of *M. crudelis* larvae between treatments (F_(1,9)_ = 1.1167, *p* = 0.32), suggesting that DGC patterns do not strongly reduce the exit of water vapor from the tracheal system in this species. Comparisons of general fluctuation patterns in water vapor output, however, differed between treatments. Larvae of *M. crudelis* in the buried treatment had significantly more peaks in water vapor output (9.2 ± 0.73) than those in the exposed treatment (2.6 ± 0.75) (*t* test, t_(8)_ = 6.29, *p* < 0.01). Specific values for transpiration rates are not provided here, as explained in the “discussion” section.

### Tracheal morphology and wettability

Larvae of *M. crudelis* have a tracheal system morphology similar to that of other studied insects. Externally, the lateral sides of the body have a series of spiracles located between pedunculated setiferous processes. Internally, tracheae with a diameter of ≈70 μm extend from the spiracles and continue to branch out, progressively decreasing in diameter. The tracheae consist of ring-like taenidia that likely provide circumferential stability and prevent the tracheae from permanently collapsing (Figures 2A and 2B). High-magnification imaging with scanning electron microscopy (SEM) revealed that the micro-sized taenidia (1.1 ± 0.1 μm in width, *n* = 5) are interspersed by regions of nanobumps (1.5 ± 0.2 μm region between taenidia, nanobump diameter of 161.3 ± 0.1 nm, *n* = 5), creating a hierarchical and rough topography (Figures 2B and 2C). The taenidia are present throughout the length of the tracheae, except for the terminal liquid-filled tracheoles, and nanobumps were observed inside tracheae with diameters as small as 2 μm.

**Figure 2:**
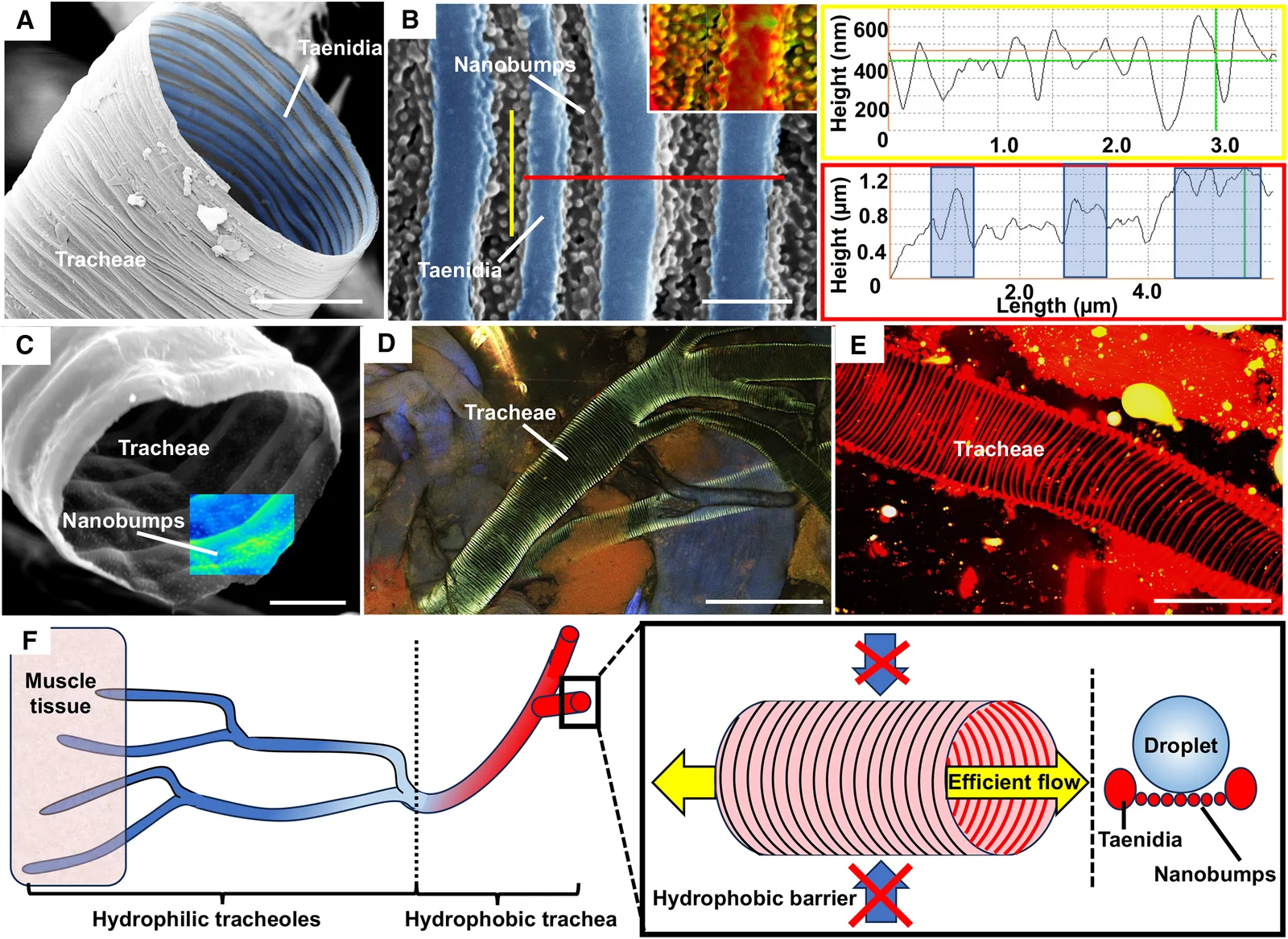
Tracheal morphology and wettability of antlion larvae (A) SEM image of a trachea showing the ridge-like taenidia (false-colored blue). (B) High-magnification SEM image showing the surface of the tracheal wall where nanobumps are located between the taenidia (height map shown in inset). The yellow and red lines correspond to surface topography measurements shown on the right of the SEM image, where the yellow graph shows nanobump roughness and red graph shows taenidia and nanobump roughness. (C) SEM image of trachea showing that nanobumps are present even in tracheae with smaller diameters (surface height map shown embedded on SEM image). (D) CLSM image of unstained tracheae showing patterns of autofluorescence that relate to the material properties and degree of sclerotization. The tracheae appeared primarily green, indicating moderate sclerotization. (E) Trachea stained with Nile Red showing strong red fluorescence, indicating its hydrophobicity. (F) Schematic showing how trachea wettability might relate to gas flow. Left image shows how the hydrophobic trachea lead to the hydrophilic tracheoles that are responsible for gas exchange in the tissues. The hydrophobicity of the tracheae likely confines the liquids to the tracheoles. The image on the right shows a hydrophobic trachea that would lessen water vapor permeability through the tracheal wall (indicated by blue arrows with the red “X”). The inside of the tracheal conduits will support the efficient flow of respiratory gases (yellow arrows) due to its potential superhydrophobicity via Cassie-Baxter theory (shown on right as a spherical droplet on the rough tracheal surface). Scale bars: (A) 10 μm, (B) 2 μm, (C) 5 μm, (D) 200 μm, and (E) 100 μm.

We investigated the material properties and wettability of tracheae with confocal laser scanning microscopy (CLSM). Unstained tracheae were first examined to understand its material properties and degree of sclerotization based on patterns of autofluorescence. Tracheae primarily exhibited green autofluorescence, indicating moderate sclerotization (Figure 2D), which likely provides flexibility for possible tracheal compressions while maintaining stability to prevent the tracheae from permanently collapsing. In contrast, tracheae stained with Nile Red displayed strong red fluorescence, notably the taenidia, clearly indicating their hydrophobicity, which was even observable through the layer of epidermal cells that surround the tracheal wall (Figure 2E). The chemical hydrophobicity of the surface combined with the hierarchical topography provides strong evidence that the inner conduits of tracheae are superhydrophobic.

## Discussion

According to the chthonic hypothesis, DGC patterns are adaptive for subterranean insects by facilitating the generation of concentration gradients of respiratory gases, which would enable efficient gas exchange in a hypoxic-hypercapnic environment.^11^ The changes in respiration patterns without corresponding changes in temperature and/or humidity (respirometer set at 25°C and 50% rh) clearly indicate that these variables, considered important for DGC patterns,^10^ were not the cause here for antlion larvae to switch to DGC. Instead, our results suggest that residing in the possibly hypoxic-hypercapnic pit resulted in the change in respiration patterns.

Although there were no significant differences in overall larval transpiration rates between treatments, the greater number of peaks in the buried treatment, where DGC patterns were observed, suggests that short bursts of water vapor might also exit the tracheal system with the bursts of CO_2_ during the *O* phase of DGC. Despite that these findings support the chthonic hypothesis, it does not mean that this hypothesis, and others, such as the hygric hypothesis, are mutually exclusive in other species, i.e., the DGC respiration pattern could be adaptive for gas exchange in hypoxic-hypercapnic environments and also conserve water.

The wettability of the tracheae might have important implications for gas exchange processes and potential dehydration. The possible superhydrophobicity of the inner tracheal conduit is understandable through the Cassie-Baxter theorem,^23^ where the presence of surface nanobumps and a hierarchical topography creates spaces that trap air, thus preventing the spreading of droplets and causing a composite solid-liquid-air interface, a phenomenon found on many biological surfaces.^24^ The superhydrophobicity of surfaces is typically demonstrated with contact angle measurements of droplets that exceed 150°. Contact angles of approximately 170° were reported for water droplets placed on the tracheal surface of adult butterflies and moths,^6^ which have a similar hierarchical topography as the tracheae of *M. crudelis* larvae. Measuring the contact angles on the inside surface of tracheae of *M. crudelis* larvae, however, is not feasible due to their small sizes, hence why Nile Red, a lipophilic stain, was used to study hydrophobicity.

The superhydrophobicity in the inner tracheal surface could serve as a barrier to prevent water droplets from entering the tracheae through the spiracles that could disrupt gas flow. A similar effect might occur at the opposite ends of the tracheae where they border the tracheoles, confining the fluids to the tracheoles, which are likely hydrophilic, thus preventing the tracheole liquids from exiting and spreading as a film along the inner surface of the tracheae. In addition, the general hydrophobicity of the tracheal wall, as shown with the Nile Red staining (Figure 1E), would lessen the loss of water vapor from the insect’s hemolymph through the tracheal wall and into the tracheae, further reducing potential desiccation (Figure 1F). Additional details of tracheae wettability should be emphasized in future studies to understand its role in gas exchange processes and possible dehydration.

Interestingly, the findings of tracheal wettability create a paradox, as the potential superhydrophobicity of the tracheal surface, in combination with their dimensions, would support rapid gas flow enabling efficient gas exchange, but also would facilitate the rapid flow of water vapor (potentially from the tracheoles) leading to dehydration. This possibility is considered due to the sizes of the tracheal conduits (diameters < 70 μm here) that would create low Knudson numbers (a dimensionless value used to assess microfluidic dynamics), indicating a slip flow regime.^4^ Therefore, the gases and liquid vapors that flow along the hydrophobic walls would be subjected to hydrodynamic slip, enabling rapid movement of gas and water vapor molecules through the conduits. Despite this conflicting scenario, water vapor loss in this study was minimal in both treatments, but the tracheal material properties would still facilitate rapid gas exchanges essential to DGC respiratory patterns.

There are several future studies that can build upon the findings presented here. For example, O_2_ concentrations in the respirometer can be manipulated to determine if respiratory patterns change from continuous to DGC, even with exposed antlion larvae. In addition, changing the porosity of the soil (i.e., changing the size of the sand particles in the pits) would affect the extent of hypoxia in the soil, which combined with fiber optic sensors could determine the specific O_2_ concentration inside the pits and at what concentration threshold the respiration patterns change. These experiments were not performed here to keep the experimental conditions similar to their natural environment. Furthermore, the respiration patterns can be examined in context of antlion life history traits, particularly when they leave a pit to build another or when they exhibit post-contact immobility in response to predators, which could provide insight into the adaptive value of switching from one pattern to another. Patterns of DGC among the many insect lineages is sporadic and primarily lacking in ancestral groups,^10^ indicating convergence, which also suggests that the evolution DGC is due to species experiencing similar selection pressures. However, not all insects with DGC patterns are subterranean; therefore, the selection pressures might be related to a general need for decoupling concentration gradients of respiratory gases rather than strictly adhering to subterranean species.

### Limitations of the study

This study provided insights into the wettability of tracheae; however, it is lacking in specific measurements of its permeability to water vapor. A more thorough and focused examination coupled with an appropriate experimental design is needed to better understand tracheae wettability and how it relates to insect gas exchange patterns, and we hope future insect respiration studies will consider this. Along with this, the present study did not directly measure O_2_ or CO_2_ levels inside the pit, which is needed to determine if the environment inside the pit is truly hypoxic-hypercapnic. The application of fiber optic sensors could be used to determine these values.

An important limitation of the study is our experimental design for transpiration measurements, which involved using an insect respirometer chamber composed of plastic. Though ideal for assessing insect gas exchange patterns, water molecules passing through the respirometer might adhere to the plastic chamber walls, affecting overall measurement values. The possible adherence of water molecules to the chamber wall, combined with the differences in flow rates between CO_2_ and water vapor molecules (due to differences in their molar masses), prevent accurate correlations between respiration and transpiration rates. Therefore, the analysis here focused only on overall differences in average transpiration rates and general fluctuation patterns of water vapor between treatments rather than specific transpiration measurement values. Future studies should consider using a smaller respirometer chamber composed of a hydrophobic material that would equally enable the flow of respiratory gases and water vapor as to better correlate these parameters.

## Resource availability

### Lead contact

Further information and requests for resources should be directed to and will be fulfilled by the lead contact, Matthew S. Lehnert (mlehner1@kent.edu).

### Materials availability

This study did not generate new or unique reagents.

### Data and code availability

• The data required for analyzing the results presented in this article can be obtained from the lead contact upon request or in the FigShare data repository: https://doi.org/10.6084/m9.figshare.33249300.
• There was no code used for this manuscript.
• Any additional information required to analyze the data reported in this manuscript is available from the corresponding author upon request.

## Acknowledgments

We thank Dr. David Ward (Kent State University) for access to the respiration equipment, Connie Kramer and Martin Walschburger Hurtado (Kent State University at Stark) for acquiring supplies, and Tori Hart and Anika Walter (Kent State University at Stark) for reading an early version of the manuscript.

## Author contributions

Conceptualization, M.S.L.; methodology, M.S.L.; investigation, M.S.L. and K.O.M.; visualization, M.S.L. and K.O.M.; supervision, M.S.L.; formal analysis, M.S.L.; writing – original draft, M.S.L. and K.O.M.; both authors reviewed and edited the final version of the manuscript.

## Declaration of interests

The authors declare no competing interests.

## STAR★Methods

### Key resources table

**Table:** 

REAGENT or RESOURCE	SOURCE	IDENTIFIER
**Deposited data**		

Raw data	Figshare data repository	https://doi.org/10.6084/m9.figshare.33249300

**Experimental models: Organisms/strains**		

*Myrmeleon crudelis*	Antlionfarms.com	N/A
*Drosophila melanogaster*	Antlionfarms.com	N/A

**Software and algorithms**		

JMP version 19	https://www.jmp.com/en/home	N/A

**Other**		

Li-Cor System	Li-Cor	N/A
Li-Cor Insect Respiration Chamber	Li-Cor	N/A

### Experimental model and study participant details

Third-instar larvae of the antlion, *Myrmeleon crudelis* Walker (Neuroptera: Myrmeleontidae), were wild caught (Gainesville, FL) or obtained from AntlionFarms.com (Pensacola, FL) and maintained in plastic containers at 22°C–24 °C and 40–50% rh. Individuals were fed fruit flies, *Drosophila melanogaster*, for sustenance, but were not fed for at least 24 h before the respiration experiments.

### Method details

#### Respiratory experiments

We measured CO_2_ output volume from antlion larvae via flow-through respirometry using a Li-Cor 6800 system (Li-Cor, Lincoln, NE, USA). The insect respiration chamber (Li-Cor, 6800–89; 11.25 cm long, 3.0 cm diameter, chamber volume of 49.9 cm^3^) was attached to the Li-Cor system and set to a flow rate of 400 μmol s^−1^, 50% rh, 25°C, and the CO_2__r was set to 400 μmol mol^−1^. Each larva had its mass (g) entered into the settings before being placed into the insect chamber and the CO_2_ output (ug g^−1^ min^−1^) was recorded every 1 s for a total of 30 min. For the experimental treatment (i.e., buried treatment), five antlion larvae were individually placed into plastic caps (diameter 1.5 cm, height 1 cm) filled with sand and allowed to construct a pit. The cap with the larva inside the pit was positioned inside the respiration chamber 24 h after initial setup and CO_2_ output levels and transpiration values were recorded. For comparison, we used a positive treatment (i.e., exposed treatment) with five different antlion individuals that consisted of exposed larvae (no pit) individually placed inside the insect chamber, and a negative treatment where the chamber was left empty. The experiments for the exposed and buried treatments were conducted in a room with the lights on. Different individuals of antlion larvae were used between the treatments to ensure independent measurements and to prevent carryover effects, such as potential stress to the antlions that could impact their breathing patterns.

#### Scanning electron microscopy

Larvae of *M. crudelis* previously stored in a −80°C freezer were dissected with scissors and insect pins to isolate cuticular regions with spiracles and to expose tracheae. The structures were placed through an ethanol dehydration series (80%, 90%, 100%, 24 h each), then allowed to air dry for at least 24 h. The specimens were then secured to an SEM aluminum stub with carbon graphite tape and sputter coated with 20 nm of platinum (150T S sputter coater, Electron Microscopy Sciences, Morgantown, PA, USA). The spiracles and tracheae were imaged at different magnifications with a JEOL 6010LV SEM (JEOL, Pleasanton, CA, USA) at 20 kV and a spot size of 50. Additional 3D images were created with InTouchScope™ SEM software. SEM images were later false-colored in Adobe Photoshop.

#### Confocal laser scanning microscopy

Tracheae were dissected from four larvae of *M. crudelis* using insect pins and dissecting scissors. Tracheae from two specimens were used as a control treatment and were left unstained, while tracheae from two other specimens were placed in an experimental treatment that included submerging the tracheae in a droplet of Nile Red-acetone stain. The droplet of Nile Red with the tracheae was allowed to completely dry (approximately 15 min), and the excess stain was washed from the sample using dH_2_0. Specimens were immediately placed on a glass slide in a droplet of molecular grade glycerol (≥99.0%, ThermoFisher Scientific) and placed under a high-resolution coverslip (12 mm diameter, thickness no. 1.5 H, Neuvitro Corporation, USA).

Samples were imaged at 10x and 20x magnifications with an Olympus FluoView 3000 confocal microscope (Olympus America Inc., Center Valley, PA, USA) using previously established protocols.^25^ Four solid state lasers with emission wavelengths of 415–485 nm (blue), 500–600 nm (green), 565–660 nm (red), and 644–744 nm (red) and excitation wavelengths of 405, 488, 561, and 640 nm were used, respectively. For the unstained treatment, the voltage and percentage laser strength, which ranged from 450–550 V and 10–15%, respectively, were adjusted independently for each laser to prevent oversaturation. The same methods were used for the tracheae stained with Nile Red, except the intense red fluorescence required adjusting the red lasers to 400 V and laser strengths between 7–10%. The number of z-slices was chosen based on the recommendations of the software, and a maximum-intensity projection was applied to each image. Images were processed in ImageJ^26^ and saved as PNGs.

### Quantification and statistical analysis

Comparisons in the length of time (s) among the different DGC phases were performed by first averaging the length of each phase per individual, the comparing the lengths of phases among individuals with an ANOVA to test for significance (*p* < 0.05). All average values are presented as the mean ± SEM. In addition, t-tests were performed to compare respiratory patterns and transpiration rates between the positive and experimental treatments. All tests were performed in JMP v.19 and the raw data are available at FigShare data repository (https://doi.org/10.6084/m9.figshare.33249300).
